# The utility of the emBODY tool as a novel method of studying complex phenomena-related emotions

**DOI:** 10.1038/s41598-022-23734-4

**Published:** 2022-11-18

**Authors:** Aleksandra M. Herman, Dominika Zaremba, Bartosz Kossowski, Artur Marchewka

**Affiliations:** Laboratory of Brain Imaging (LOBI), Nencki Institute of Experimental Biology, Polish Academy of Sciences, Pasteur 3, 02-093 Warsaw, Poland

**Keywords:** Psychology, Emotion

## Abstract

Bodily sensations are one of the major building blocks of emotional experience. However, people differ in their ability to recognise and name their emotions, especially those in response to complex phenomena such as climate change or the COVID-19 pandemic. Therefore, we investigated whether the bodily sensation maps (BSMs) approach can be employed to study emotions related to phenomena that are likely to evoke various, and perhaps even conflicting, emotions in people. Using a unique topographical self-report method—the previously established emBODY tool, 548 participants marked where in the body they feel sensations (activations and deactivations) when they experience distinct emotions (e.g. happiness) and when they think about different phenomena, namely climate change, COVID-19 pandemic, war, nature, friends, and summer holidays. We revealed maps of bodily sensations associated with different emotions and phenomena. Importantly, each phenomenon was related to a statistically unique BSM, suggesting that participants were able to differentiate between feelings associated with distinct phenomena. Yet, we also found that BSMs of phenomena showed some similarity with maps of emotions. Together, these findings indicate that the emBODY tool might be useful in uncovering the range of emotions individuals experience towards complex phenomena.

## Introduction

Early theories of emotion posited that motivationally relevant stimuli elicit autonomic changes, which then lead to emotional experience^1,2^. Modern theories of embodied emotion (for example^3,4^) extend these early formulations arguing that the occurrence and interpretation of somatic response to the emotional stimuli are necessary for the emergence of experiential feelings of anger, anxiety or joy. Additionally, processing of emotional information involves a (partial) reexperience of an emotion^4^. Thus, feelings and sensations in the body are one of the major building blocks of emotional experience^5^.

Importantly though, according to the Conceptual Act Theory^6,7^ bodily sensations we experience in different circumstances, undergo our individual interpretations (i.e., are constructed) depending on external and internal triggers and our individual past experiences within the constraints of the language. Thus, the emotions (e.g., fear, anger, happiness, etc.) are the *meanings* that we assign to the bodily states we feel using a specific language and the cultural norms the language is embedded in. Thus, the same bodily sensations might be interpreted differently by various individuals. For example, bodily signals such as one’s face getting red, general jitteriness, and increased heart rate might be assigned as anxiety by one person in given circumstances or as excitement by another person. Similarly, people might show individual differences in how emotions are represented in the body although previous studies showed that at the population level, self-reported bodily representations of various emotions are universal across cultures^8^.

Emotions arise when an event is relevant to one’s values and concerns^9^; however, many events and phenomena of the modern world may elicit a range of different emotions. For example, emotional evaluations of the risks related to climate change can be dependent on different cognitive appraisals. The appraisal of climate change as an anthropogenic risk that endangers only nature results in a different emotional experience (grief) than the appraisal of it as a risk to human civilisation (anxiety, terror)^10^. These appraisals can be held by the same person at the same time leading to a simultaneous experience of various distinct emotions. Indeed, a growing body of evidence indicates that people report a range of emotional responses linked to different aspects of climate change, such as sadness, grief, distress, despair, disgust, anger, fear, anxiety, helplessness and hopelessness but also hope or fascination^11–18^. Similarly, in the context of the COVID-19 pandemic, one may consider the need for social distancing and work from home as a threat to their current way of life or more spare time for leisure instead of commuting. These appraisals will be related to different emotions related to the pandemic (increased depression versus lower stress/increased relaxation, respectively)^19^. Yet, people may be lacking linguistic tools to express what they feel regarding complex phenomena such as climate change or COVID-19 which can hinder understanding of one's emotional states^20^. Therefore, as complex global phenomena, such as climate change or COVID-19 pandemic elicit a range of different, hard to name and perhaps even conflicting emotions, they are challenging to be studied with declarative self-report methods. The use of common self-report methods of studying emotions, where individuals indicate to what extent positive and negative emotional words describe their feelings, or choose a graphical (e.g., faces, manikins) or numeric representation of emotions on valence and arousal (or other) dimensions that match their feelings (e.g.^21–23^), may be suboptimal to study some phenomena-related emotions, as provided (emotional words) options might be insufficient to cover the whole plethora of emotions one experiences regarding a given issue or, in case of sensitive social topics, they may lead to an experimenter expectancy effect.

Thus, to study subjective feelings related to such complex phenomena as climate change or COVID-19, we may need an indirect method of emotional assessment. The development of the emBody tool^8,24^ provides a new method of emotional reports that allows participants to *draw* where in the body they feel activity changes in response to different emotions. The emotion-specific bodily sensation patterns seem to be universal across cultures^8^ and across different types of emotional stimuli (verbal vs nonverbal) and emotion induction techniques (such as emotional videos and guided emotional imagery)^24^. The technique is intuitive and has been successfully used in children^25^ and psychiatric populations^26^. Therefore, we employed the emBODY tool to (1) investigate whether we can use the bodily sensation maps (BSMs) approach to study emotions related to phenomena that are likely to evoke various, and perhaps even conflicting, emotions in people (namely climate change, COVID-19, war, friends, summer holidays, nature), (2) to check where in the body people map their sensations related to global phenomena such as climate change, COVID-19 pandemic or war, (3) as well as to assess to what extent the BSMs of phenomena are similar to BSMs of distinct emotions. We included several phenomena to establish whether participants can indicate topographic representation of each phenomenon and whether these maps are different from each other or generalisable across all phenomena. Some selected phenomena were intended to differ in valence (e.g. war vs friends). COVID-19 and climate change were selected as important global phenomena that are likely to evoke strong emotions in the general population, but climate change is a constant slow process, while COVID-19 pandemic was a more sudden and time-specific phenomenon.

## Methods

### Participants

Here we employed emBODY methodology^8,24^ with a total of 620 individuals completing the study (age 31.00 ± 10.55, range: 18–83, 87% females). All participants had to be 18 or older, currently live in Poland and be regular Polish language users. Participants were recruited online, via social media (Twitter, Facebook) as well as local mailing lists and word of mouth. Importantly, we always advertised our research as a study aimed to improve understanding of where in their bodies people place emotional reactions associated with various emotional states and phenomena, without naming climate change or COVID-19 specifically (the study was conducted during the third wave of the COVID-19 pandemic in Poland in spring 2021). In this way, we wanted to target a population with a variety of attitudes towards those issues (i.e. both people who are strongly concerned with climate change as well as those who might be climate change deniers). All participants provided informed consent. No incentive was provided for participants. The procedures were approved by the SWPS Ethical committee in Poznań (approval no. 2021-52-12). All procedures were conducted following the Declaration of Helsinki. The study is a part of a larger project financed by Norway Grants No. 2019/34/H/HS6/00,677 which we conduct in collaboration with SWPS University in Poznań; thus, ethical approval was obtained for several parts of the project at once from the SWPS Ethical committee (as at Nencki Institute there is no ethics board that approves human research).

### Procedures

**Figure 1:**
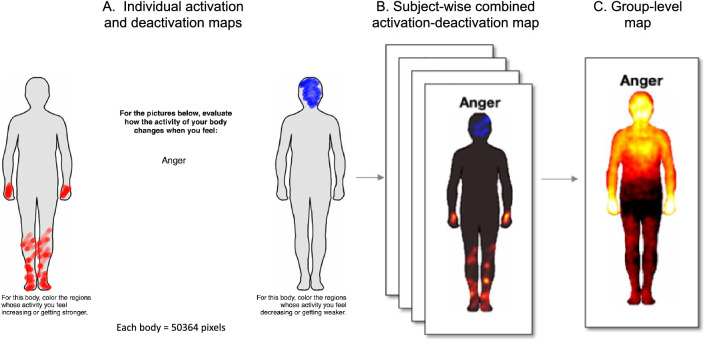
emBODY tool. (**A**) Representation of a single trial showing individual activations and deactivation maps for Anger. (**B**) The subject-wise analysis involves merging individual activation and deactivation maps into a single image and smoothing. Images preprocessed that way undergo visual inspection. (**C**) Finally, individual images are subjected to a one-sample t-test to produce group-level maps. These maps are used in subsequent analyses.

Data collection took place online. Demographic and other self-report information were gathered using LimeSurvey (https://www.limesurvey.org) while data regarding the topographical representation of emotions were acquired using the emBODY tool^24^. Firstly, volunteers provided informed consent to participate in the study and answered basic demographic questions (age, gender, place of residency, education). Next, the task followed (Fig. 1), whereby participants were shown two human silhouettes on a computer screen with an emotion or a phenomenon word placed between the two body outlines. In the first part, participants saw emotion words and used the mouse to colour the bodily regions which activity they felt increasing or getting stronger on the left body, and regions which activity they felt decreasing or getting weaker on the right body when experiencing that emotion. Following previous studies^8,24^, 14 emotion words (fear, anger, disgust, sadness, happiness, surprise, anxiety, love, depression, contempt, pride, shame, jealousy and neutral state) were presented sequentially, the order being shuffled randomly for each participant. In the second part, participants saw 6 words describing phenomena (climate change, COVID-19, war, friends, summer holidays, nature) in a shuffled order and were asked to colour in body regions which activity they felt increasing and decreasing (as above) when thinking about each phenomenon. The emotions mapping was always completed first as we knew from previous studies that participants are able to perform this task. In this way, we also wanted to introduce this idea of focusing what one feels in their body, before completing the maps of phenomena. Therefore, in total, participants had 20 body sensation maps to complete (14 emotional maps and 6 phenomena maps). There was no time limit to complete the task but typically it took approximately 15 min.

Finally, participants answered a set of questions related to climate change and COVID-19 concerns (see Table 1 for details). These questions assessed the subjective intensity of emotions experienced in relation to climate change and COVID-19 as well as the personal experience of them. Participants answered the questions on a 5-point scale ranging from ‘I strongly disagree’ (1) to ‘I strongly agree’ (5).

**Table 1:** Questions related to climate change and Covid-19 pandemic used in the study. Each question was rated on a Likert scale from 1 (I strongly disagree) to 5 (I strongly agree).

Aspect	Index	Item
Climate change	CC1	I Experience strong emotions related to climate change
	CC2	My strong emotions associated with climate change affect my mood and/or daily functioning
	CC3	I have been directly affected by climate change
	CC4	I know someone who has been directly affected by climate change
Covid-19	C1	I experience strong emotions related to the COVID-19 pandemic
	C2	My emotions associated with the COVID-19 pandemic affect my mood and/or daily functioning
	C3	I have been directly affected by the COVID-19 pandemic
	C4	I know someone who has been directly affected by the COVID-19 pandemic

### Data analysis

#### Quality control and group-level analysis

We explored the topographic representation of each emotion across participants using the Nummenmaa and co-workers’ methodology^8,24^. Specifically, we first reconstructed BSMs from data collected during the web survey. For each participant, a single BSM comprising 50,364 pixels was obtained for each emotion and phenomenon, with activation and deactivation coded as positive and negative values, respectively. Responses outside the body area were masked. As a single mouse click reached several hundred pixels, we used a Gaussian disk to smooth the coloured areas, thereby incorporating spatial dependency information into the maps to prevent the exaggeration of embodiment. Next, we screened for sufficient completion rates: Participants leaving more than mean—2.5 SDs (M = 18.79, SD = 2.06) of bodies untouched were removed from the sample. Moreover, we manually screened individual maps for anomalous responses (e.g., writing or drawing symbols).

For each emotion, statistically significant activations and deactivations were assessed by means of mass univariate *t*-tests: a one-sample *t*-test against zero was performed for each pixel within a BSM, resulting in a statistical *t*-map. Following past studies employing the emBODY approach^8,24^, to account for multiple comparisons, each statistical map was then thresholded using the False Discovery Rate (FDR) correction (*ɑ* = 0.05). The FDR correction was also used in all subsequent analyses.

#### Similarity

To quantify the similarity between BSMs of different emotions and phenomena, we computed a pixel-wise Spearman-correlation-based similarity matrix between each pair of the group-level maps (obtained in the one-sample *t*-tests analysis). Additionally, to establish to what extent the BSMs of emotions in the current study replicate past findings, we run a seperate similarity analysis with the BSMs based on data from the previous^8^ large-scale study (N = 3,085). We computed a similarity index (Spearman’s correlation coefficient) between the corresponding BSMs from the current and the reference study.

#### Classification

Following past studies, we tested whether different emotions and phenomena are associated with statistically distinct bodily patterns using statistical pattern recognition with linear discriminant analysis (LDA)^24,27^. Dimensionality was first reduced to 30 components with principal component analysis and then LDA was applied. The accuracy of the model was determined with fivefold cross-validation where classifiers were trained to discriminate all stimuli from each other (complete classification). To statistically test classifier accuracy against chance level, the cross-validation was run iteratively 100 times^24,27^.

## Results

### Exclusions and demographics

Based on an insufficient BSM completion rate (min 13 maps completed), 65 individuals were excluded from the analyses. Additional 7 individuals were excluded following initial screening and visual inspection of individual maps (e.g., writing or drawing symbols). Therefore, 548 individuals were included in the final analysis (age 30.75 ± 10.56).

**Figure 2:**
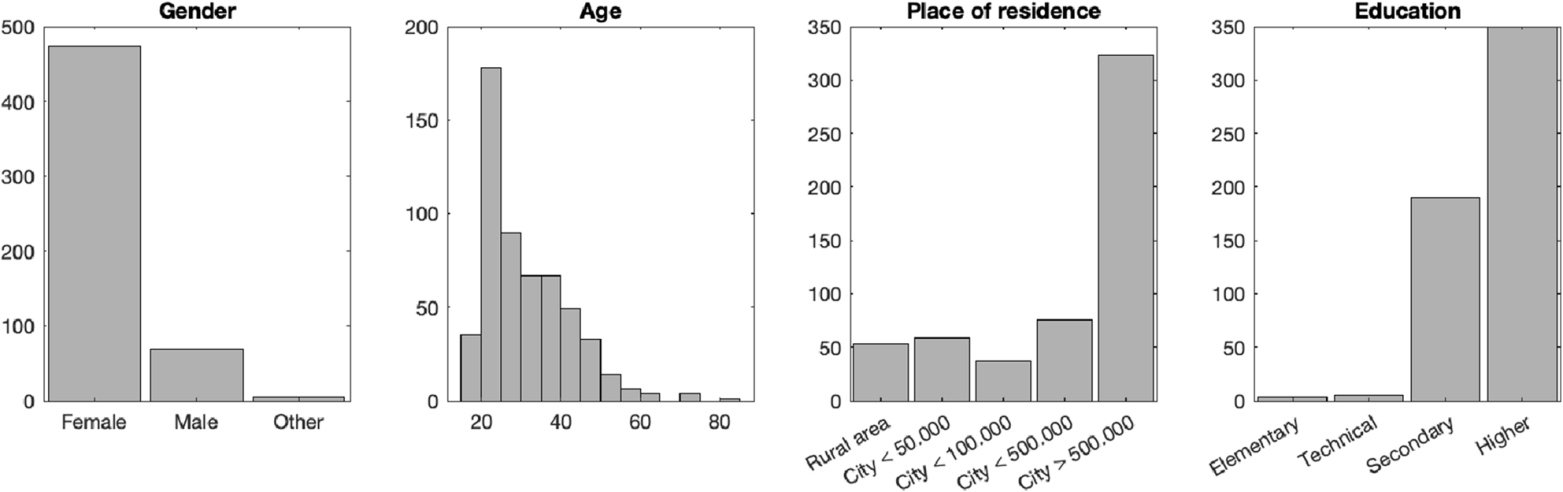
Demographic information about the study sample (N = 548) included in the final analyses.

Information regarding the final demographics is included in Fig. 2. Overall, the current sample consisted of mainly young adult females with higher education, from large urban areas. Although our sample cannot be considered representative of the population, this is a very typical representation of an opportunity sample collected online and is highly comparable to samples studied in previous studies using the emBody tool^8,24^.

**Figure 3:**
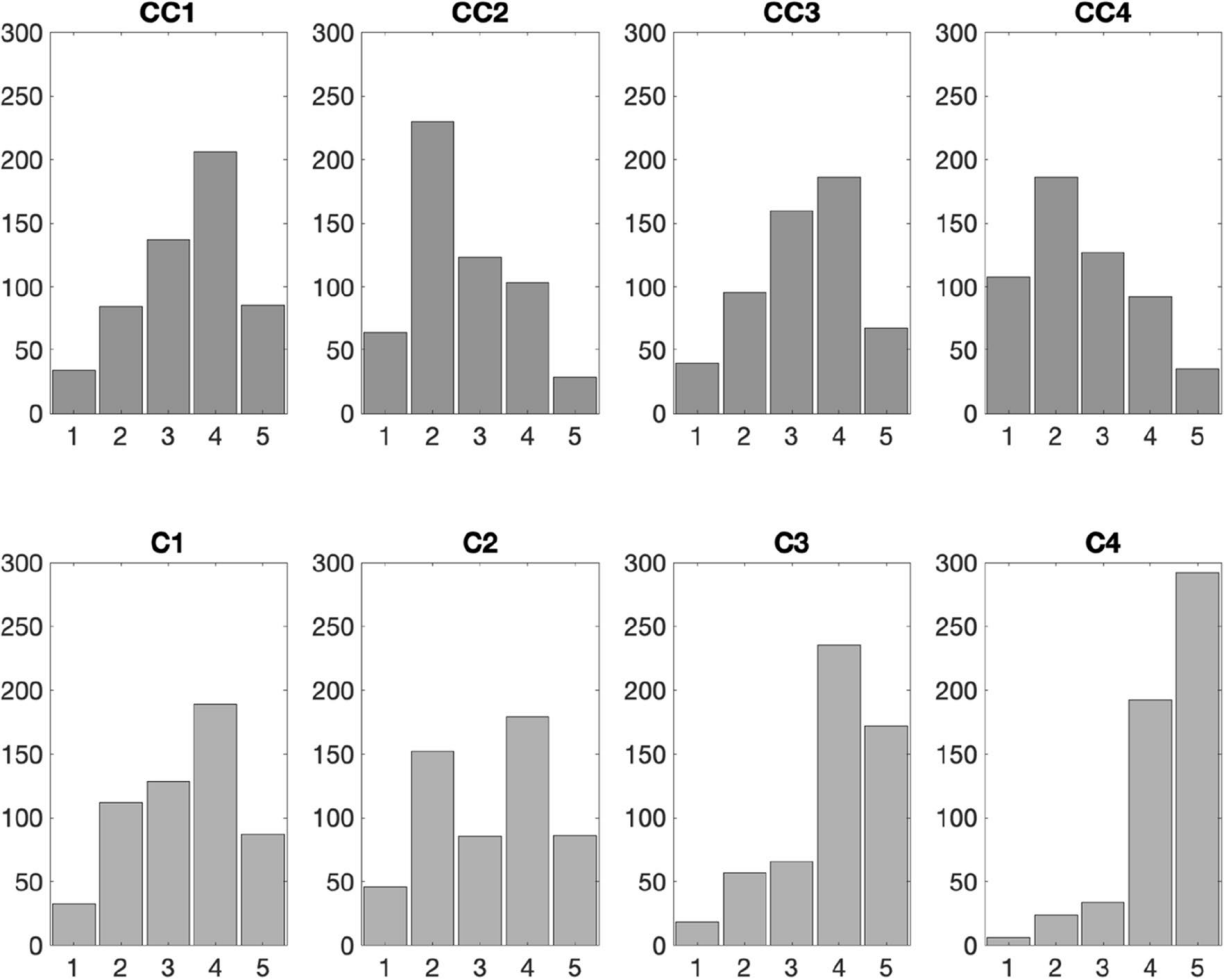
Histograms showing the distribution of responses to the climate change (top row) and COVID-19 (bottom row) related questions. For comparison purposes, the histograms are organised in such a way that corresponding questions related to climate change and COVID-19 are presented in the same column. The questions were rated on a Likert scale from 1 = I strongly disagree to 5 = I strongly agree. For the exact questions’ text, see Table 1.

Figure 3 shows the distribution of responses to the questions regarding climate change and COVID-19 concern (also see Table 1 for the full questions wordings). Overall, the participants tended to agree (i.e., answered 4 or 5 on the scale, 53%) with the statement that they experience strong emotions related to climate change (CC1), however, fewer (24%) claimed that these emotions affect their daily mood and/or daily functioning (CC2). 46% agreed that they had been negatively affected by climate change (CC3), yet only 23% agreed that they knew someone who had been directly affected by climate change (CC4). These answers suggest that our sample was rather aware of the climate change problem and some people seemed to be emotionally affected by it.

Interestingly the distribution of responses regarding emotional reaction to COVID-19 (C1), was very similar to the one in relation to climate change, with around 50% of participants reporting experiencing strong emotions to COVID-19 pandemic. In contrast to climate change responses, more people (48%—twice as many as in response to climate change) agreed with the statement that their strong emotions affect their mood and/or daily functioning. The vast majority (74%) said they were directly affected by the pandemic (CC3) and 88% knew someone who had been directly affected by it (CC4, skewness <− 1.5). Therefore, although a comparable number of participants in our sample reported experiencing strong emotions with respect to both climate and pandemic, participants tended to be more affected by the pandemic.

### Emotion- and phenomena-specific body sensation maps

**Figure 4:**
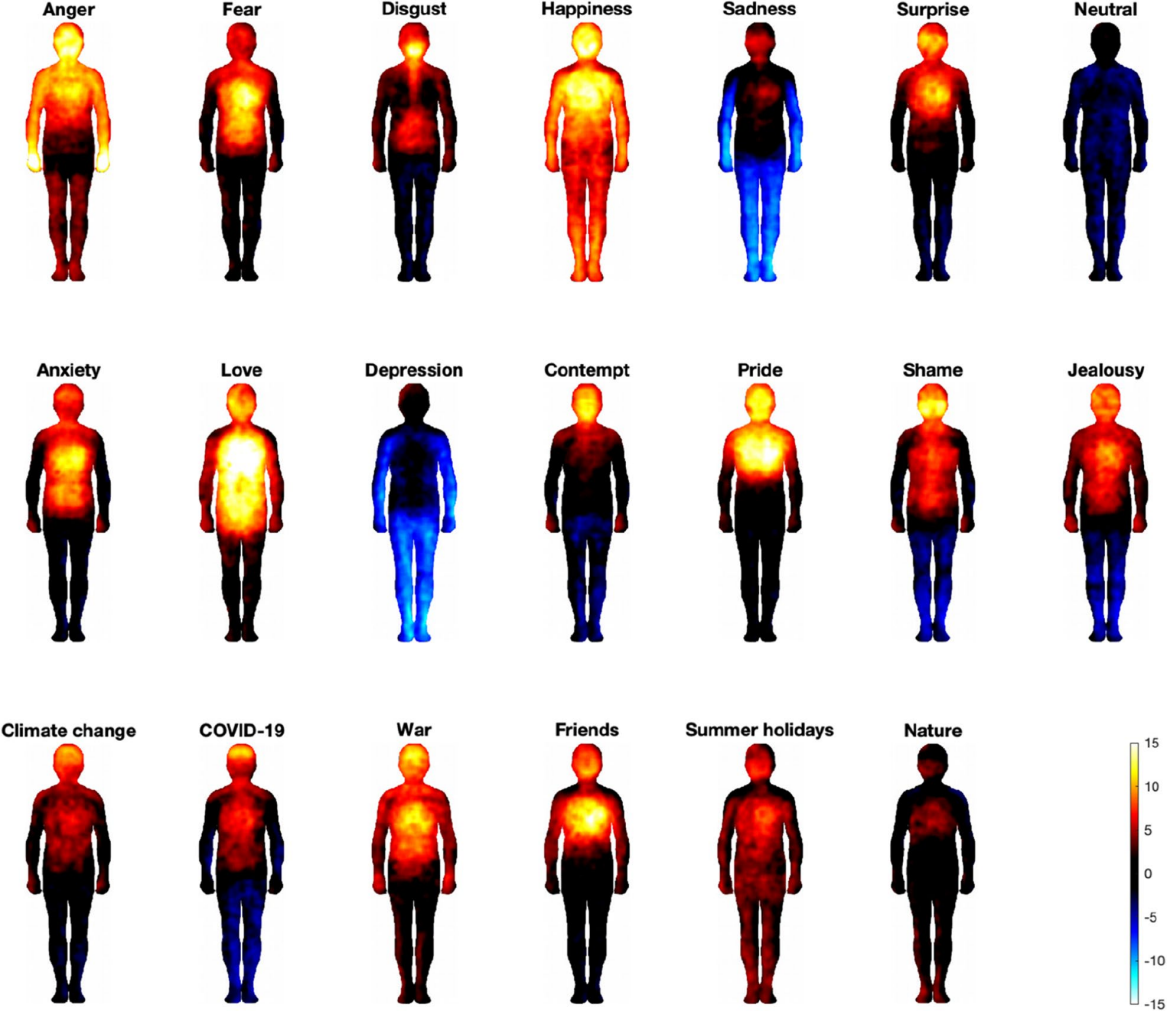
Bodily topography of basic (Upper) and complex (Middle) emotions and sensations related to phenomena (Lower). The body maps show regions whose activation increased (warm colours) or decreased (cool colours) when feeling each emotion or thinking of each phenomenon (*p* < 0.05 FDR corrected; t > 2.95). The colour bar indicates the t-statistic range.

Different emotions were associated with statistically clearly separable bodily sensation maps (Fig. 4). Sadness, depression and neutral state were predominantly associated with deactivations, particularly intensive in limbs, while other emotions were mainly associated with activations that were found predominantly in the chest and head. Shame, jealousy and contempt were also linked to deactivations in legs. Shame also showed a characteristic activation in the cheeks. Disgust showed a very specific pattern of activations along the digestive system. We also observed clearly separable BSMs of phenomena: While nature was related to weak activations in the head, chest and partly legs, summer holidays were related to stronger activations throughout the body, war and friends were related to robust activation in the head, chest and arms, with war-linked activations also expanding to hands and abdomen. Finally, while both COVID-19 and climate change were related to strong activations in the head, chest, and abdomen, COVID-19 also showed strong deactivations in the legs, while climate change showed activations in the hands.

### Similarity across body sensations maps

**Figure 5:**
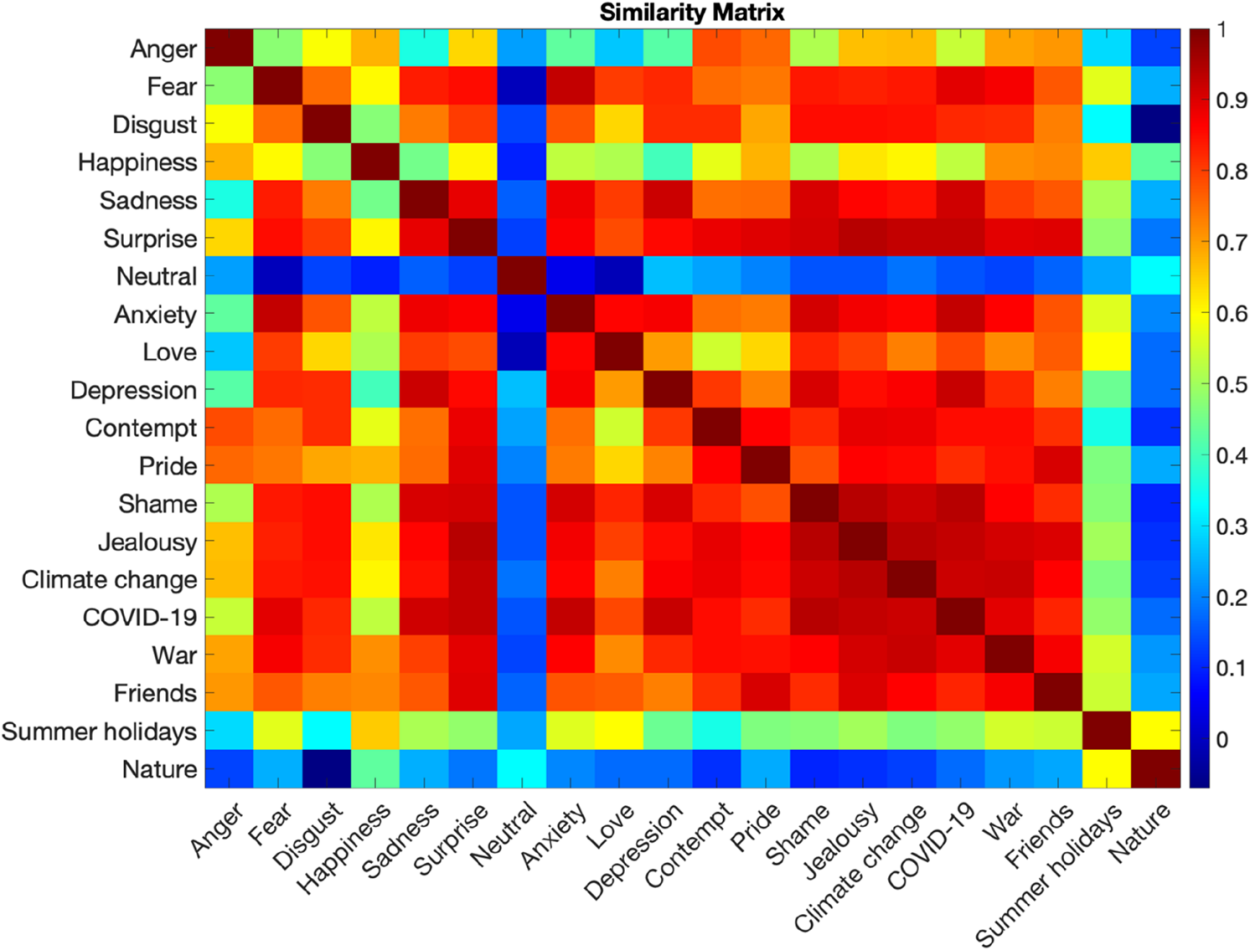
The similarity between each pair of emotions and phenomena (Spearman’s correlation). Each cell in the matrix represents a pairwise correlation coefficient between two body sensation maps. The colour bar indicates the r-statistic range.

To quantify the similarity between discrete BSMs of emotions and climate change, we run Spearman’s correlations analysis, between each pair of group-level BSMs. The full correlation matrix between each pair of BSMs is presented in Fig. 5. Overall, the greatest correlations (i.e. similarity) between the pairs of emotions were present between jealousy and surprise (*r* = 0.94), jealousy and shame (*r* = 0.94) and anxiety and fear (*r* = 0.93). Regarding phenomena, the highest similarity was present between the BSM of climate change and war (*r* = 0.92). Climate change BSM showed very high similarity (*r* > 0.80) with several different emotions, namely: fear, disgust, sadness, surprise, anxiety, depression, contempt, pride, shame, and jealousy. A similar pattern was also observed for the BSMs of COVID-19 and war. Summer holidays and nature BSMs showed only weak to modest similarity with emotions and phenomena BSMs (*r’s* < 0.60^28^).

**Figure 6:**
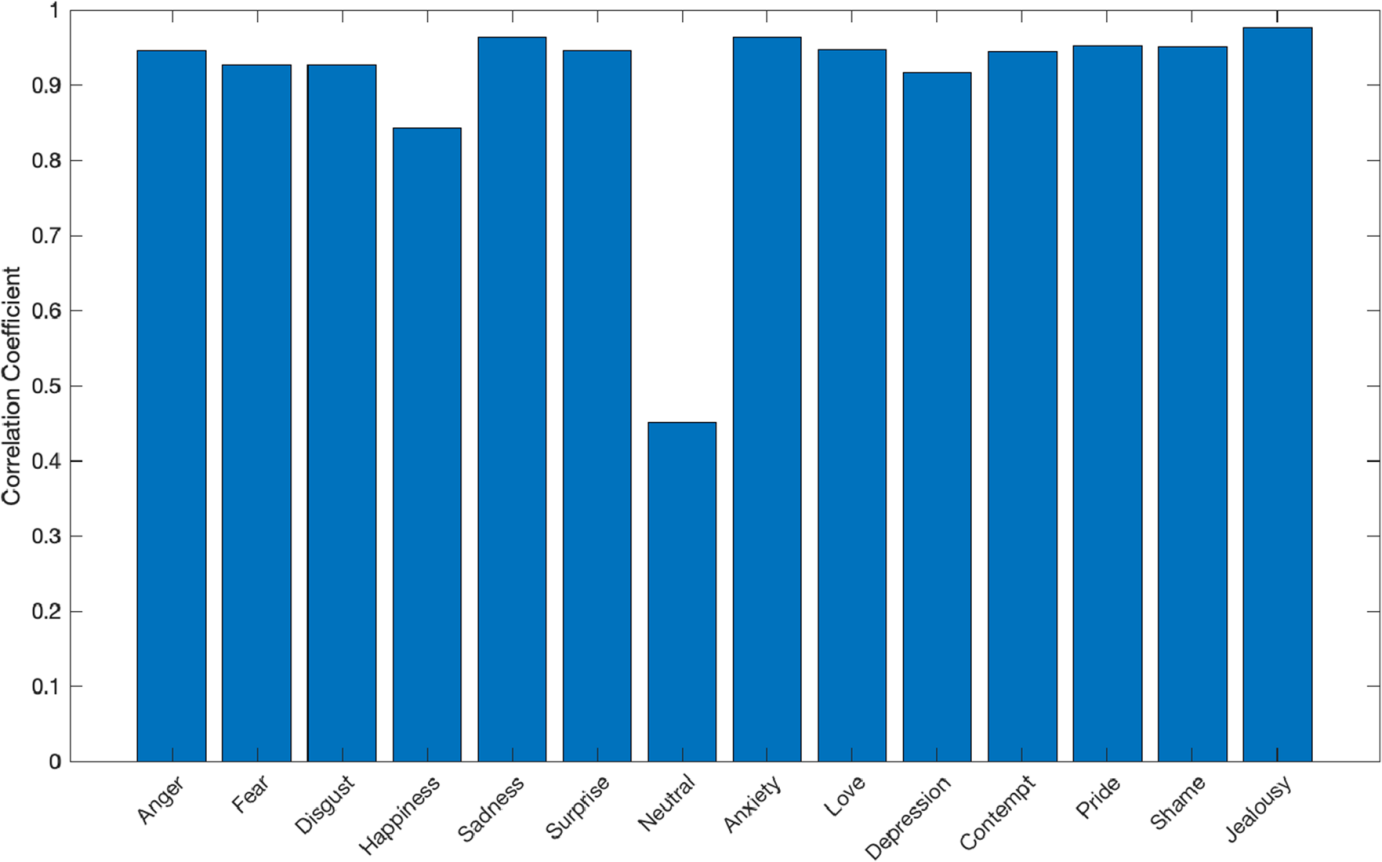
The similarity (Spearman’s correlation coefficient) between the BSMs of corresponding emotions in the current and previous study^8^.

Additionally, as presented in Fig. 6, the corresponding BSMs of emotions in the current study and previous report^8^ showed a high similarity (r ≥ 0.84) for all but one BSM. The Neutral state showed lower similarity (r = 0.45), which is probably due to the low number of pixels painted for this BSM.

### Classification

**Figure 7:**
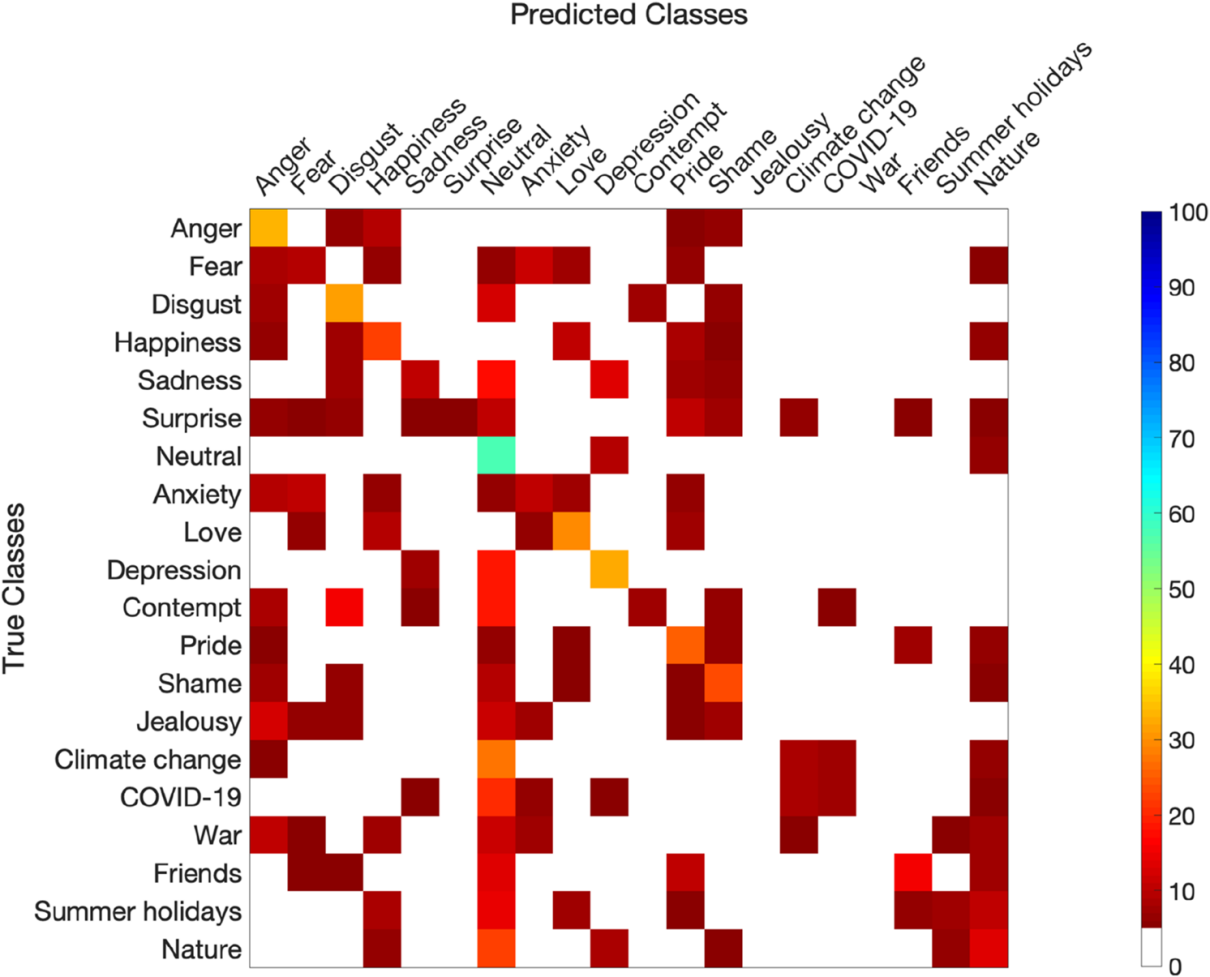
Confusion matrix between predicted and true classifications. White spaces represent classifications below chance level (5%).

Results of the LDA indicate that the BSMs can be correctly classified with an overall accuracy rate of 17.74% (± 0.15), which is above the chance level (5%). The confusion matrix (Fig. 7) indicates that a neutral emotional state was classified the most accurately (57.24%), followed by anger (33.15%), depression (32.65%) and disgust (31.82%). Jealousy and war BSMs were correctly classified in just above 2% of the cases, which is below the chance level.

Notably, a few emotions were misclassified, specifically, fear (classified accurately 9.43% of the time) was more often classified as anxiety (10.47%), while anxiety as fear (10.18% and 11.80%, respectively), confirming the results from the similarity analysis and suggesting that these two emotions either produce very similar bodily sensations, indeed, or people confuse the two together. Additionally, contempt was equally often classified as disgust (7.19% and 7.75%, respectively).

## Discussion

The current study employed the emBODY tool to (1) investigate whether we can use the bodily sensation maps (BSMs) approach to study emotions related to phenomena that are likely to evoke various, and perhaps even conflicting, emotions in people, (2) to check where in the body people map their sensations related to global phenomena such as climate change, COVID-19 pandemic or war, (3) as well as to assess to what extent the BSMs of phenomena are similar to BSMs of distinct emotions. Overall, our results indicate that the body sensations mapping task is understandable to participants and is suitable to study emotions related to different phenomena as indicated by distinct localisation and characteristics of BSMs linked to phenomena used in current study. Specifically, we showed that even complex phenomena, such as climate change, evoke sensations that participants experience in specific locations in the body. Furthermore, we revealed that the BSMs of important global phenomena (i.e. climate change, COVID-19 pandemic, and war) showed high similarity with many unique emotions, specifically with fear, disgust, sadness, surprise, anxiety, depression, contempt, pride, shame, and jealousy, thus, suggesting that at the population level, people may experience a whole variety of different, mainly negative, emotions in relation to these phenomena^11–17,29,30^. Below, we discuss our findings in detail.

Firstly, regarding BSMs of basic and complex emotions, our results replicate previous findings^8,24^, with BSMs of the current study showing a striking similarity to the previously obtained results. This is despite the fact that our sample was smaller than those in the previous studies and we conducted the study in Polish (previously conducted in English). These results support the notion that body maps of emotions are culturally universal at the population level and it also gives us confidence in the novel aspect of the current study: the BSMs of phenomena.

Secondly, participants localised their sensations related to important current phenomena (i.e., climate change, COVID-19 and war) as activations in the head, chest and abdomen. The strongest activation observed in the head may mean that these phenomena evoke high-level mental processing^31^. Indeed, past research on mapping subjective feelings showed that many cognitive processes such as thinking, being conscious, attending, memorising, reasoning, inferring or estimating are consistently related to sensations in the head area^32^. Past research also indicated that positive emotions are typically felt as activations of the head/face and the chest^31^, which is also in agreement with the results from the present study. This pattern is also observed regarding negative emotions but to a lesser extent and intensity. Negative emotions also tend to be represented more as deactivations, particularly in the limbs^31^. Activations in the heart/chest area may be associated with the increase in heart rate or faster breathing/holding one’s breath, commonly experienced in many emotions regardless of their valence. Indeed, previous work has shown that increased heart rate is related to both positive (e.g. happiness, joy and anticipated pleasure) and negative (e.g. anger, anxiety, fear and sadness) emotions^33^. COVID-19, and to a lesser extent climate change, BSMs also showed deactivations in the legs, which may reflect a feeling of immobility and avoidance, resembling the deactivation found in the depression and sadness maps.

Therefore, in case of complex phenomena-evoked emotions, activations in the chest and deactivations in legs may suggest that individuals experience a range of different emotions towards climate change, and often these may be conflicting (e.g. anxiety about the future and hope for change^17^). The findings from the similarity analysis are also in line with the notion that complex phenomena investigated in the present study are linked to a variety of emotions in people. We revealed that the pattern of sensations observed for climate change, COVID-19, and war showed high similarity (*r* > 0.80) with a range of BSMs of emotions, namely fear, disgust, sadness, surprise, anxiety, depression, contempt, pride, shame, and jealousy. Interestingly, out of all phenomena, climate change BSM showed the highest similarity to the BSM of war, suggesting that both likely evoke similar, plausibly negative, emotions. Indeed, a growing body of evidence indicates that people report a range of emotional responses linked to different aspects of climate change, such as sadness, grief, distress, despair, disgust, anger, fear, anxiety, helplessness and hopelessness but also hope or fascination^11–17^. Similarly, research has shown that people report various emotions regarding the COVID-19 pandemic, both positive (e.g., relaxation, happiness) and negative (e.g., stress, anxiety, depression), and that these emotions can co-occur^29,30^.

Despite such striking similarities between the BSMs of important global phenomena and emotions, emotions and phenomena are associated with statistically distinct bodily patterns, as indicated with the LDA. The findings regarding the BSMs of emotions are largely in line with past research^24,27^. Specifically, the BSMs of neutral state, anger and happiness are consistently reported as emotions with the highest classification accuracy, confirming their unique bodily sensation pattern. Importantly, beyond confirming these findings, we also showed that the BSMs of feelings related to complex phenomena also show distinct bodily topography. This was true for climate change and COVID-19 maps, but not for the map of war, which had classification accuracy below the chance level. Possibly, it is easier for individuals to indicate their emotions to current phenomena (the present study was conducted in spring 2021, during the 3rd wave of the pandemic in Poland and before the breakout of the war in Ukraine, a country located next to Poland), hence the low discrimination for the BSM of war. Future research could investigate whether the BSMs of COVID-19 or war are time-sensitive and differ according to socio-political events.

Some limitations of our study merit comment. This study was conducted in an opportunity sample of internet users recruited online via social media, mailing lists and word of mouth. Although we did have a broad age range (18–83) in our sample, the majority were young women with higher education living in big cities. Likely, these demographics may explain a relatively high concern for climate change in our sample and a large proportion of individuals who reported experiencing strong emotions related to climate change^34^. In the future, it would be important to replicate current findings in a representative European sample regarding age, gender, education and socioeconomic status. Additionally, to assess the general attitudes towards climate change and COVID-19 pandemic and to keep the study short (under 20 min), we created our own questions of attitudes, which were not previously validated. Finally, as people may be lacking linguistic tools to express what they feel regarding complex phenomena such as climate change or COVID-19, we decided not to use any direct questions about emotions people feel towards these phenomena. Future studies may also ask participants to rate different emotions associated with these complex phenomena on a Likert scale allowing for a direct comparison between verbal reports and BSMs.

To our knowledge, this is the first study to explore the topographical maps of phenomena-related emotions. Future studies could investigate the effect of psychological distance (an indicator of how close or distant people feel from the issue) towards distinct phenomena on the topography/intensity of drawing on the bodies (i.e. the intensity of emotions)^35,36^. Future studies could also investigate whether the body maps of phenomena are culturally universal, as in the case of body maps of emotions^8^ or are they different (e.g., does the climate change BSM differ across cultures/countries that vary in pro-ecological support or those that are likely to be affected by climate change sooner vs later?).

## Conclusions

Beyond replicating previous research on BSMs of basic and complex emotions, our study employed the emBODY tool to investigate whether the body sensations mapping approach can be applied to study emotions related to important global phenomena. Our findings not only suggest that participants are able to differentiate bodily sensations related to distinct phenomena, but also show that we can use the similarity between emotion-phenomena pairs to investigate what the underlying emotions may be. We conclude that the emBODY tool is valid to study emotions related to complex global phenomena.

## Data Availability

Data and analyses code is available here: https://osf.io/vkpm6/?view_only=022c8a40c5ab4508a9dddf28dcf8da57.

## References

[1] James W (1884). II—What is an emotion?. Mind.

[2] Lange, C.G. The mechanism of the emotions. Class. Psychol*.* 672–684 (1885).

[3] Damasio AR (1989). Time-locked multiregional retroactivation: A systems-level proposal for the neural substrates of recall and recognition. Cognition.

[4] Niedenthal PM (2007). Embodying emotion. Science.

[5] Zhou P, Critchley H, Garfinkel S, Gao Y (2021). The conceptualization of emotions across cultures: A model based on interoceptive neuroscience. Neurosci. Biobehav. Rev..

[6] Barrett LF (2006). Are emotions natural kinds?. Perspect. Psychol. Sci..

[7] Barrett LF (2014). The conceptual act theory: A Précis. Emot. Rev..

[8] Volynets S, Glerean E, Hietanen JK, Hari R, Nummenmaa L (2020). Bodily maps of emotions are culturally universal. Emotion.

[9] Zeelenberg M, Nelissen RMA, Breugelmans SM, Pieters R (2008). On emotion specificity in decision making: Why feeling is for doing. Judgm. Decis. Mak..

[10] Böhm G, Pfister H-R (2017). The perceiver’s social role and a risk’s causal structure as determinants of environmental risk evaluation. J. Risk Res..

[11] Albrecht G (2007). Solastalgia: The distress caused by environmental change. Australas. Psychiatry.

[12] Clayton S, Karazsia BT (2020). Development and validation of a measure of climate change anxiety. J. Environ. Psychol..

[13] Cunsolo A, Ellis NR (2018). Ecological grief as a mental health response to climate change-related loss. Nat. Clim. Chang..

[14] Ellis NR, Albrecht GA (2017). Climate change threats to family farmers’ sense of place and mental wellbeing: A case study from the western Australian Wheatbelt. Soc. Sci. Med..

[15] Leiserowitz, A. A., Maibach, E., Roser-Renouf, C., Feinberg, G. & Rosenthal, S. *Climate change in the American mind*. (University of Washington, 2018).

[16] Minor, K. *et al.* Greenlandic perspectives on climate change 2018–2019: Results from a national survey. (2019).

[17] Wang S, Leviston Z, Hurlstone M, Lawrence C, Walker I (2018). Emotions predict policy support: Why it matters how people feel about climate change. Glob. Environ. Change.

[18] Pihkala P (2022). Toward a taxonomy of climate emotions. Front. Clim..

[19] Gurvich C (2021). Coping styles and mental health in response to societal changes during the COVID-19 pandemic. Int. J. Soc. Psychiatry.

[20] Barrett LF (2017). How emotions are made the secret life of the brain.

[21] Bradley MM, Lang PJ (1994). Measuring emotion: The self-assessment manikin and the semantic differential. J. Behav. Ther. Exp. Psychiatry.

[22] Broekens J, Brinkman W-P (2013). AffectButton: A method for reliable and valid affective self-report. Int. J. Hum. Comput. Stud..

[23] Mehrabian A (1995). Framework for a comprehensive description and measurement of emotional states. Genet. Soc. Gen. Psychol. Monogr..

[24] Nummenmaa L, Glerean E, Hari R, Hietanen JK (2014). Bodily maps of emotions. Proc. Natl. Acad. Sci. U. S. A..

[25] Hietanen JK, Glerean E, Hari R, Nummenmaa L (2016). Bodily maps of emotions across child development. Dev. Sci..

[26] Torregrossa LJ (2019). Anomalous bodily maps of emotions in Schizophrenia. Schizophr. Bull..

[27] Lyons N (2021). Bodily maps of emotion in major depressive disorder. Cognit. Ther. Res..

[28] Taylor R (1990). Interpretation of the correlation coefficient: A basic review. J. Diagn. Med. Sonogr..

[29] Adikari A (2021). Emotions of COVID-19: Content analysis of self-reported information using artificial intelligence. J. Med. Internet Res..

[30] Moroń M, Biolik-Moroń M (2021). Trait emotional intelligence and emotional experiences during the COVID-19 pandemic outbreak in Poland: A daily diary study. Pers. Individ. Differ..

[31] Novembre G, Zanon M, Morrison I, Ambron E (2019). Bodily sensations in social scenarios: Where in the body?. Plos One.

[32] Nummenmaa L, Hari R, Hietanen JK, Glerean E (2018). Maps of subjective feelings. Proc. Natl. Acad. Sci. U. S. A..

[33] Kreibig SD (2010). Autonomic nervous system activity in emotion: A review. Biol. Psychol..

[34] Lewis GB, Palm R, Feng B (2019). Cross-national variation in determinants of climate change concern. Env. Polit..

[35] Chu H, Yang JZ (2019). Emotion and the psychological distance of climate change. Sci. Commun..

[36] McDonald RI, Chai HY, Newell BR (2015). Personal experience and the ‘psychological distance’ of climate change: An integrative review. J. Environ. Psychol..

